# Judgment of Learning Reactivity for Emotional Faces

**DOI:** 10.3390/bs16081443

**Published:** 2026-08-21

**Authors:** Morgan O. Lemler, Daniel M. Bialer, Minyu Chang

**Affiliations:** 1 Department of Psychology, Trinity University, San Antonio, TX 78212, USA; mlemler@trinity.edu; 2 Department of Psychological Science, University of Arkansas, Fayetteville, AR 72701, USA; dbialer@uark.edu

**Keywords:** judgment of learning reactivity, emotional face recognition, elaborative processing account, changed-goal hypothesis, cross-race effect

## Abstract

Making judgments of learning (JOLs) can directly modify future memory, which is termed JOL reactivity. Recently, JOLs were found to produce positive reactivity for negative faces but not for positive faces. The finding was interpreted as support for the changed-goal hypothesis, which posits that making JOLs highlights item differences in learning difficulty, thereby prompting people to become more performance-oriented and to focus more on items perceived as easier (e.g., negative faces) at the cost of items perceived as harder (e.g., positive faces). While face emotion was manipulated in a mixed-list design in a prior study, we used a pure list of either positive or negative faces during encoding, which minimizes incentives to change learning goals. We replicated the finding of positive reactivity for negative faces and null reactivity for positive faces even in the pure-list design. Additionally, this pattern was consistent across same-race and cross-race faces and persisted when faces were tested with neutral rather than emotional expressions, demonstrating the robustness of the finding. Our results suggest that the emotion-related difference in JOL reactivity for face recognition cannot be explained by the changed-goal hypothesis. Instead, the current findings may be explained by the elaborative processing account, which assumes that making JOLs engages more elaborative processing than people spontaneously do. Because positive faces are spontaneously processed more elaborately than negative faces during encoding, this account posits that JOLs should provide a weaker reactivity for positive faces than for negative faces.

## 1. Introduction

Judgments of learning (JOLs) are a common metamemory measurement in which learners estimate how likely they are to remember the encoded item in the future during or immediately after encoding (Rhodes, 2015). Accumulating research has shown that making JOLs not only monitors memory processes, but also directly modifies subsequent memory performance, a phenomenon known as JOL reactivity (Ingendahl et al., 2025). Here, positive reactivity means that making JOLs improves memory, whereas negative reactivity refers to the effect that making JOLs impairs memory. 

Currently, several theoretical accounts have been proposed to explain JOL reactivity, and the mechanisms underlying JOL reactivity remain debated. One of the most widely examined explanations is the changed-goal hypothesis (Mitchum et al., 2016; for a more comprehensive discussion of the existing theoretical accounts of JOL reactivity, please see Chang & Brainerd, 2025; Ingendahl et al., 2025). The changed-goal hypothesis suggests that making JOLs heightens individuals’ awareness that not all items will be equally remembered, and thus, individuals tend to switch from a master-oriented goal to a performance-oriented goal. Namely, rather than trying to memorize all the items, individuals will focus more on learning the ones they deem easier to remember at the cost of those deemed harder. Therefore, this hypothesis predicts that making JOLs should produce negative reactivity for items perceived as harder to remember and positive reactivity for items perceived as easier to remember. This prediction was supported by evidence of negative reactivity for semantically unrelated word pairs, but positive or null reactivity for semantically related word pairs (Janes et al., 2018; Mitchum et al., 2016; Undorf et al., 2024). 

While some studies found that the difference in JOL reactivity between related and unrelated pairs occurred only in a mixed-list design but not a pure-list design (Janes et al., 2018; Mitchum et al., 2016), other studies found that such a difference arose in both mixed-list and pure-list designs (Maxwell & Huff, 2023; Tauber & Witherby, 2019). The mixed-list design is central to the changed-goal hypothesis because participants are clearly aware of the differences between easier items (e.g., related pairs) and harder items (e.g., unrelated pairs) in such a design, which ultimately prompts a change in learning goals. In contrast, in a pure-list design, where participants study only related pairs or only unrelated pairs, the differences in item difficulty are not discernible, which should minimize the motivation for goal shifting. Therefore, the finding that JOL reactivity still arises in a pure-list design challenges the changed-goal hypothesis. 

While the majority of existing JOL reactivity studies have relied on verbal stimuli, a few studies have extended this line of research to non-verbal visual stimuli (e.g., Chang et al., 2026; Guo et al., 2025; Shi et al., 2023; Tang et al., 2025). For instance, Chang et al. (2026) recently reported positive JOL reactivity in recognition for both cross-race and same-race faces. In that connection, it is well-documented that cross-race faces are harder to recognize than same-race faces, which is known as the cross-race effect (CRE; Meissner & Brigham, 2001; Sporer, 2001; Young et al., 2012). Moreover, it has also been found that cross-race faces receive lower JOLs than same-race faces, indicating that the former is perceived as harder to remember than the latter (Chen & Zhu, 2019; Hourihan et al., 2012). Therefore, the changed-goal hypothesis should predict that participants will focus more on the easier, same-race faces at the cost of the harder, cross-race faces, resulting in positive reactivity for the former but negative reactivity for the latter. However, this prediction is clearly not supported by the positive reactivity found for both cross-race and same-race faces (Chang et al., 2026).

Nevertheless, Guo et al. (2025) recently reported supporting evidence for the changed-goal hypothesis by testing JOL reactivity for emotional faces. In their study, participants studied a mixed list of positive, neutral, and negative faces, and they either made a JOL or not after viewing each face. Additionally, Guo et al. manipulated JOL condition (JOL vs. No-JOL) within participants in Experiment 1 and between participants in Experiment 2. Based on prior research findings that the impact of JOLs was highest for negative pictures, followed by positive and then neutral pictures (Hourihan, 2020), Guo et al. predicted that negative faces would be perceived as easier to remember than positive or neutral pictures. Therefore, the changed-goal hypothesis should predict that making JOLs would prompt participants to focus more on negative faces at the cost of positive or neutral faces, thus producing positive reactivity for negative faces and negative reactivity for positive faces. 

Guo et al. found a robust pattern across both Experiments 1 and 2 that (a) JOLs were higher for negative than positive faces, and both were higher than for neutral faces; and (b) there was positive JOL reactivity for negative faces but null reactivity for positive or neutral faces. Thus, they concluded that their findings aligned with the changed-goal hypothesis: Participants assessed negative faces as being easier to remember and thus focused more on the negative faces than positive or neutral ones, leading to positive reactivity for the former but not for the latter. However, uncertainties remain regarding the theoretical implications of these findings. As acknowledged by those authors, the absence of negative reactivity for the positive and neutral pairs is not fully consistent with the changed-goal hypothesis, because the changed goal should impose a cost on the (perceived) harder items, resulting in negative reactivity. Thus, it remains ambiguous whether the emotion-related difference in JOL reactivity can be taken as evidence supporting the changed-goal hypothesis. 

## 2. The Current Study

In the current study, we evaluated the theoretical mechanism for Guo et al.’s (2025) novel finding of the emotion-related difference in JOL reactivity. Firstly, as discussed previously, a mixed-list design is central to the changed-goal hypothesis. Therefore, to further test the changed-goal hypothesis, we employed a pure-list design in which participants studied only positive faces or only negative faces. Such a design should not engage participants in comparative processing about the difference in (perceived) difficulty between positive and negative faces. As a result, there should be minimal motivation to change learning goals to prioritize items perceived as easier (negative faces) at the expense of items perceived as harder (positive faces). Accordingly, the changed-goal hypothesis should predict no reactivity for either positive or negative faces in the pure-list design. 

Secondly, we used both same-race and cross-race faces as memory stimuli, such that we recruited both White and Black participants, and we presented both White and Black faces during encoding. This design was motivated by the prior finding that the CRE appeared to be moderated by emotional expressions (Gwinn et al., 2015; Johnson & Fredrickson, 2005). While we (Chang et al., 2026) recently found that JOLs produce positive reactivity for both same-race and cross-race faces, that finding was restricted to neutral faces. Therefore, we sought to replicate that finding with emotional faces to assess the generalizability of the finding. 

Thirdly, while Guo et al. (2025) and Chang et al. (2026) both used the same face images across study and tests, we used faces with positive and negative expressions at study but faces with neutral expressions at test. This design was informed by prior face recognition research suggesting that using identical face images across study and tests may conflate face identification with image matching, because recognition for unfamiliar faces depends heavily on low-level image features (Burton, 2013; Collin et al., 2022; Hancock et al., 2000). Therefore, we tested the same facial identities with different expressions to (a) make sure participants’ performance reflected their memory for facial identity rather than their ability to match specific facial images, and (b) examine whether the emotion-related difference in JOL reactivity persisted when the same identities appeared with neutral expressions at test.

## 3. Method

### 3.1. Participants 

The sample included 164 participants recruited through Prolific (https://www.prolific.com/, accessed on 2 November 2025). An a priori power analysis was conducted using the WebPower package (Zhang & Mai, 2018) in R version 4.2.2. Based on the effect size η_p_^2^ = .18) reported by Guo et al. (2025; Experiment 2) and an expected power (1–β) of .80, a minimal sample size of 74 is needed to detect a JOL Condition × Emotion interaction in a planned mixed analysis of variance (ANOVA). Notably, this power analysis was based on the JOL Condition × Emotion interaction of primary interest, rather than on the full factorial structure of the current study. Compared to Guo et al.’s design, the current study treated study emotion as a between-participant rather than within-participant factor and also included participant race as a between-participant factor and stimulus race as a within-participant factor. Additionally, our study was conducted remotely rather than in person. Given these differences in design, we adopted a conservative recruitment strategy and recruited more than twice the minimum sample size indicated by the power analysis. We also evenly distributed the participant quotas so that half of the sample would be White participants while the other half would be Black participants.

All participants received monetary compensation ($2.99 or £2.25) for completing the study. All participants were located in the U.K. or U.S., were aged between 18 and 30, were fluent in English, had no literacy difficulty, and had an approval rate of >90% in their past Prolific participation. Five participants’ data were removed due to poor data quality because they failed >50% of attention checks (>50% of JOL responses in the JOL condition or >50% of box-checking in the no-JOL condition). An additional four participants were removed because they self-identified as neither Black nor White. The final sample included 155 participants (*M*_age_ = 26.63, *SD*_age_ = 3.04, 77 female), with 78 in the JOL condition and 77 in the no-JOL condition, and with 74 Black participants (36 studied positive faces and 38 studied negative faces) and 81 White participants (38 studied positive faces and 43 studied negative faces).

### 3.2. Materials

The materials consisted of 36 happy face images, 36 angry face images, and 72 neutral face images selected from the Chicago Face Database (CFD; Ma et al., 2015). The faces in each emotion category (happy, angry, neutral) were evenly distributed across four demographic groups: Black women, Black men, White women, and White men. All faces were selected based on the clarity of emotional expression and overall photograph quality. Because some models’ emotional expressions were more ambiguous than others, the first and third authors of the paper individually reviewed all stimuli, and any faces that caused doubt in perceived emotion were replaced with mutually agreed-upon, unambiguous alternatives. 

During the study phase, participants viewed either 36 happy faces or 36 angry faces, and all participants viewed only neutral faces during the test phase. The 36 happy and 36 angry faces shared the same facial identities, and the happy faces were all smiling with their mouths closed. The 72 neutral faces included 36 faces that shared the same facial identities as the studied faces (i.e., targets) and 36 that were completely new (i.e., distractors). Each distractor was selected to match a target based on physical appearance (e.g., face shape, hairstyle, hair type, hair color, and eye color). 

### 3.3. Procedure

The experiment was administered via Qualtrics. A 2 (JOL condition: JOL, no-JOL) × 2 (Study emotion: angry, happy) × (Stimulus race: White, Black) × 2 (Participant race: White, Black) mixed factorial design was used, with JOL condition, emotion, and participant race being between-participant factors and stimulus race being the within-participant factor. Participants were randomly assigned to either the JOL condition or the No-JOL condition, and all participants completed one study–buffer–test cycle. In the study phase, participants viewed 36 faces with either happy expressions or angry expressions for two seconds each. After each face was presented for two seconds, participants in the JOL condition had four seconds to rate how likely they were to remember the face on a later memory test using a scale slider from 0 (not likely at all) to 100 (totally likely). Following the procedure in our prior studies (Chang & Brainerd, 2023, 2024, 2025), participants in the no-JOL condition were given four seconds to click an empty check box, and after that, the program automatically advanced to the next face. The box-checking task was used in the no-JOL condition because it (a) imposes minimal cognitive demands and does not require explicit self-monitoring, (b) approximately matches the motor response required in the JOL condition, and (c) provides an attention check that is particularly useful in unsupervised online testing. 

After completing the study phase, participants completed a buffer task wherein they solved as many simple math problems as possible within two minutes. Participants then entered the test phase, in which they viewed 72 faces one at a time. All 72 test faces were displayed with neutral expressions, with 36 sharing the same facial identities as the 36 emotional faces presented during the study phase (i.e., targets) and the other 36 being completely new faces (i.e., distractors). Participants responded to each face by clicking either the “Yes” button if they remembered previously viewing the face during the study phase or the “No” button if they did not. Participants were explicitly informed that faces could appear with different emotional expressions at study and test and were instructed to base their recognition decisions on facial identity rather than expression match.

## 4. Results

All ANOVAs and follow-up *t* tests were conducted with the afex (Singmann et al., 2015) and emmeans (Lenth, 2018) packages in R version 4. 2. 2. The *p* values in the follow-up *t* tests were corrected for multiple comparisons using the Benjamini–Hochberg procedure (Benjamini & Hochberg, 1995). One 2 (Study emotion: positive, negative) × 2 (Stimulus race: Black, White) × 2 (Participant race: Black, White) mixed ANOVA was conducted for JOLs. Additionally, five 2 (Study emotion: positive, negative) × 2 (JOL condition: JOL, No-JOL) × 2 (Stimulus race: Black, White) × 2 (Participant race: Black, White) mixed ANOVAs were conducted for recognition accuracy, discrimination (*d*′), response criterion (*C*), hits, and false alarms, respectively. Recognition accuracy was defined as hits for targets and correct rejections for distractors. *d*′ was calculated as *z*(Hits) − *z*(False Alarms), and *C* was calculated as −0.5 × [*z*(Hit) + *z*(False Alarm)]. Hits or false alarms of 1 were corrected to (*N* − 1)/*N*, and hits or false alarms of 0 were corrected to 1/*N*, where *N* is the number of recognition test trials. 

Assumptions were examined for all six ANOVAs using the performance package in R (Lüdecke et al., 2021). Mauchly’s tests of sphericity revealed no violation of the sphericity assumption across all ANOVAs, and the inspection of residual Q-Q plots indicated no substantial departures from normality. Levene’s tests were conducted within each of the four Stimulus Race × Study Emotion combinations, which indicated that the homogeneity of variance assumption was satisfied across all ANOVAs with only two exceptions: The assumption was violated in the White-happy-face condition in the ANOVA for JOLs, *F*(1, 41) = 4.74, *p* = .035, and in the White-angry-face condition in the ANOVA for false alarms, *F*(1, 68) = 3.68, *p* = .016. Because our sample sizes were reasonably balanced and ANOVAs are generally robust to heteroscedasticity under balanced designs (Blanca et al., 2018), the two ANOVAs were retained in the paper. The full output for all ANOVAs and follow-up tests is available at https://osf.io/uz4ef (accessed on 20 July 2026). 

### 4.1. Results for JOLs

**Figure 1:**
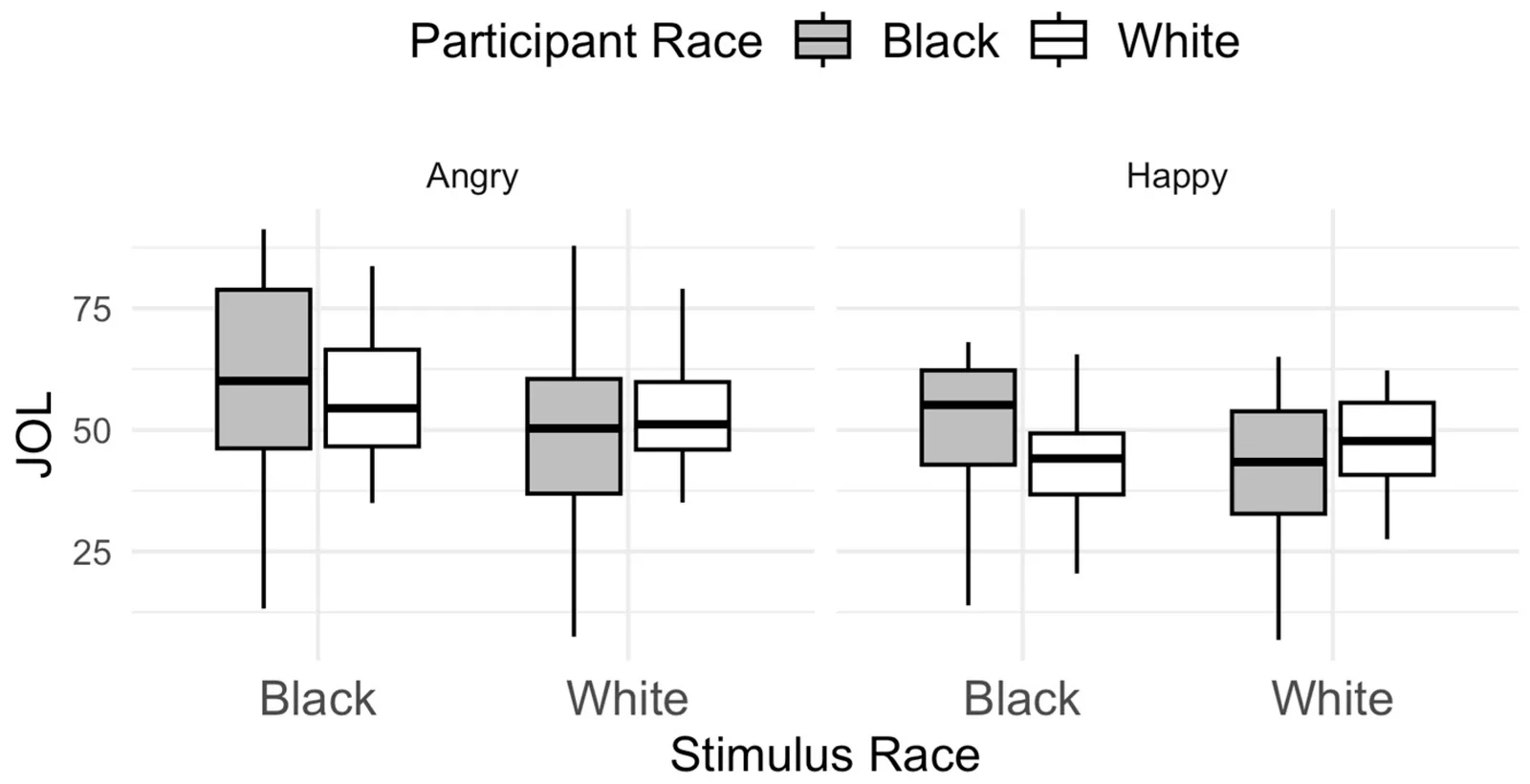
Judgment of learning (JOL) data. Note. Boxes represent the interquartile range (IQR), horizontal lines indicate medians, and whiskers extend to the most extreme values within 1.5 × IQR of the first and third quartiles.

A 2 (Study emotion: positive, negative) × 2 (Stimulus race: Black, White) × 2 (Participant race: Black, White) mixed ANOVA was conducted for JOLs. There was a main effect of study emotion, *F*(1, 74) = 6.25, *MSE* = 483.20, η_p_^2^ = .08, *p* = .015, and a main effect of stimulus race, *F*(1, 74) = 16.64, *MSE* = 483.20, η_p_^2^ = .18, *p* < .001. As shown in Figure 1, happy faces (*M* = 45.99, *SD* = 14.13) received lower JOLs than angry faces (*M* = 54.83, *SD* = 18.88), and White faces (*M* = 47.74, *SD* = 16.65) received lower JOLs than Black faces (*M* = 52.17, *SD* = 17.08).

There was also an interaction between stimulus race and participant race, *F*(1, 74) = 25.14, *MSE* = 48.85, η_p_^2^ = .25, *p* = .015. Follow-up *t* tests revealed that Black participants rated Black faces as easier to remember (*M* = 54.99, *SD* = 19.32) than White faces (*M* = 44.83, *SD* = 19.60), *t*(74) = 6.45, *d* = .52, *p* < .001. Meanwhile, White participants rated Black and White faces as comparably memorable (*M*s = 49.34 vs. 50.66), *t*(74) = −.66, *d* = −.10, *p* = .512.

### 4.2. Results for Recognition

**Figure 2:**
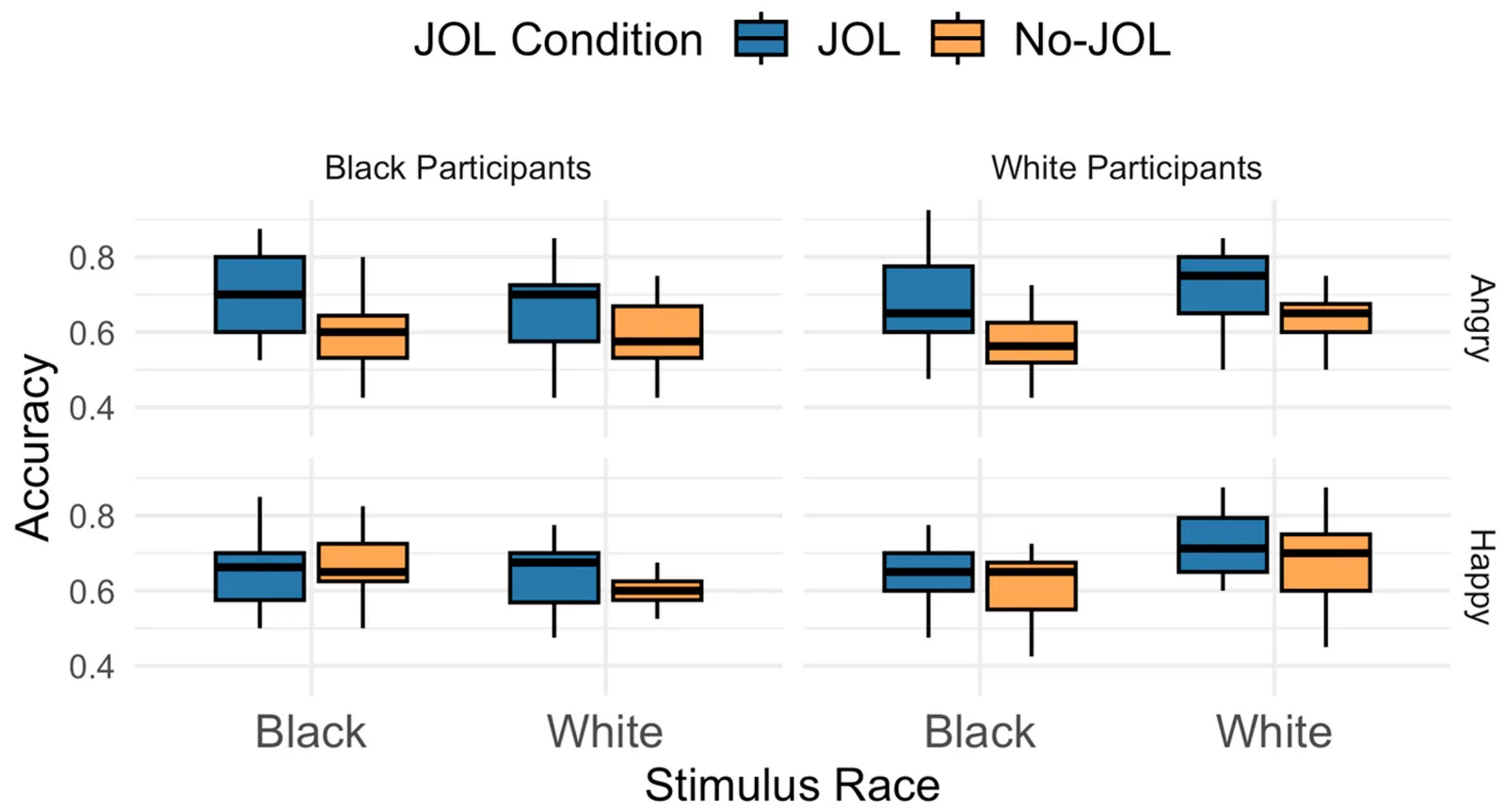
Face recognition accuracy data. Note. Boxes represent the interquartile range (IQR), horizontal lines indicate medians, and whiskers extend to the most extreme values within 1.5 × IQR of the first and third quartiles.

To begin, a 2 (Study emotion: positive, negative) × 2 (JOL condition: JOL, No-JOL) × 2 (Stimulus race: Black, White) × 2 (Participant race: Black, White) mixed ANOVA was conducted for recognition accuracy, with stimulus race being a within-participant factor while JOL condition, study emotion, and participant race were between-participant factors. There was a main effect of JOL condition, *F*(1, 144) = 17.34, *MSE* = .01, η_p_^2^ = .11, *p* < .001, and an interaction between JOL condition and study emotion, *F*(1, 144) = 3.91, *MSE* = .01, η_p_^2^ = .03, *p* = .0499. As shown in Figure 2, participants who made JOLs while studying angry faces had higher recognition accuracy on the subsequent recognition test than those who did not make JOLs (*M*s = .68 vs. .60), *t*(144) = 4.24, *d* = .81, *p* < .001. However, making JOLs did not significantly impact recognition accuracy for happy faces (*M*s = .67 vs. .64), *t*(144) = 1.59, *d* = .31, *p* = .115. Additionally, there was an interaction between participant race and stimulus race, *F*(1, 144) = 33.24, *MSE* = .01, η_p_^2^ = .19, *p* < .001. Participants had higher recognition accuracy for same-race faces than cross-race faces. Black participants were more accurate for Black than for White faces (*M*s = .66 vs. .63), *t*(144) = 2.51, *d* = .28, *p* = .013, and White participants were more accurate for White than for Black faces (*M*s = .69 vs. .62), *t*(144) = 5.73, *d* = .68, *p* < .001. 

Second, the same 2 (Study emotion: positive, negative) × 2 (JOL condition: JOL, No-JOL) × 2 (Stimulus race: Black, White) × 2 (Participant race: Black, White) mixed ANOVA was conducted for discrimination (*d*′). There were main effects of JOL condition, *F*(1, 144) = 14.72, *MSE* = .71, η_p_^2^ = .09, *p* < .001, and of stimulus race, *F*(1, 144) = 5.88, *MSE* = .28, η_p_^2^ = .04, *p* = .017, as *d*′ was higher in the JOL condition than the No-JOL condition (*M*s = 1.20 vs. .83) and was higher for White faces than for Black faces (*M*s = 1.10 vs. .93). There was also an interaction between participant race and stimulus race, *F*(1, 144) = 33.33, *MSE* = .28, η_p_^2^ = .19, *p* < .001, such that Black participants had higher *d′* for Black faces than for White faces, *t*(144) = 2.31, *d* = .27, *p* = .022, and White participants had higher *d′* for White faces than for Black faces, *t*(144) = 5.95, *d* = .72, *p* < .001. 

**Figure 3:**
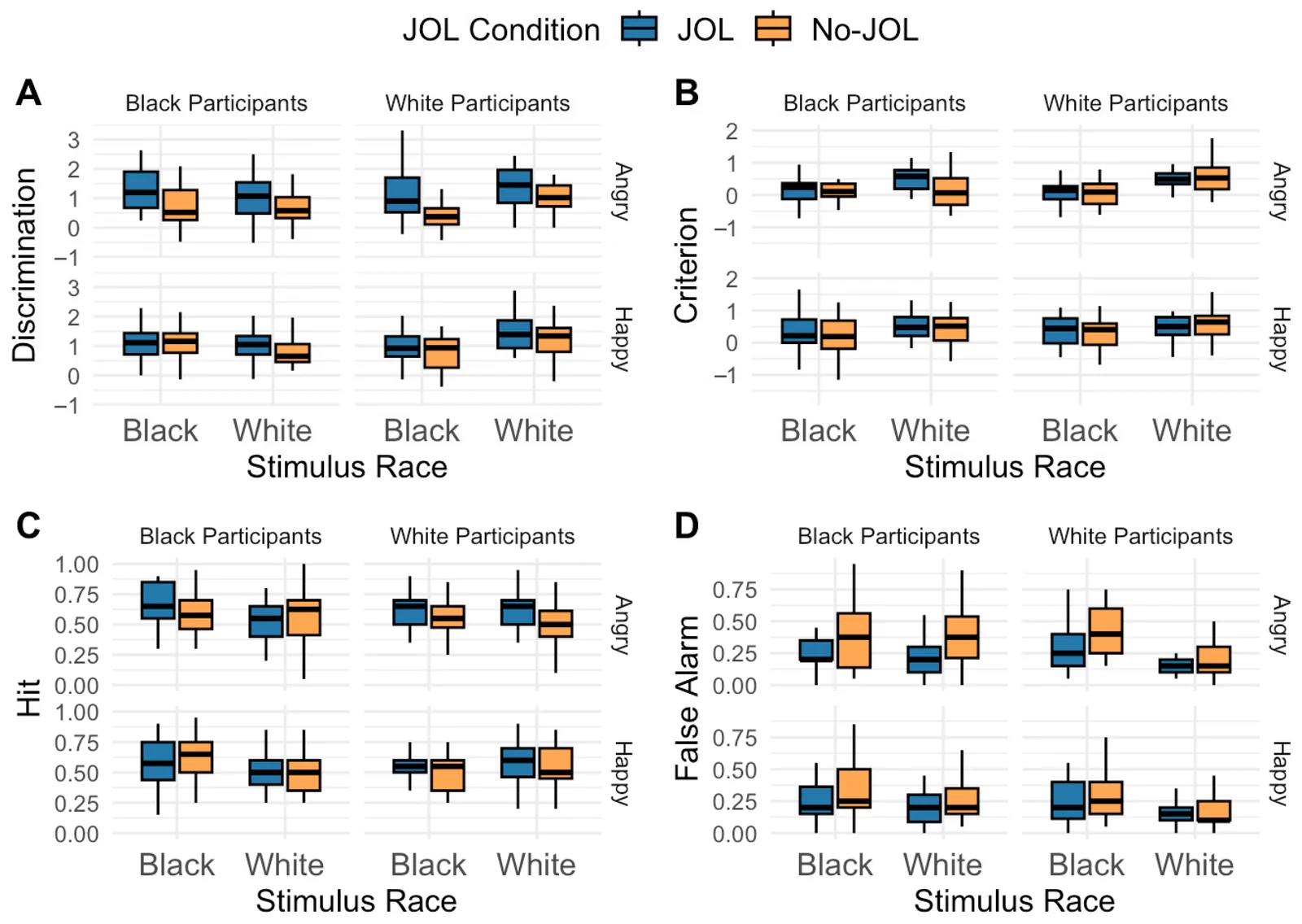
Face recognition discrimination, response criterion, hit, and false alarm data. Note. Panel (**A**) = discrimination. Panel (**B**) = response criterion. Panel (**C**) = hits. Panel (**D**) = false alarms. Boxes represent the interquartile range (IQR), horizontal lines indicate medians, and whiskers extend to the most extreme values within 1.5 × IQR of the first and third quartiles.

Although the JOL Condition × Study Emotion interaction did not reach statistical significance, *F*(1, 144) = 2.87, *MSE* = .71, η_p_^2^ = .02, *p* = .092, we still conducted the planned follow-up analyses for this interaction based on the prior findings. The follow-up *t*-tests revealed a similar pattern as for recognition accuracy, as *d*′ for angry faces were significantly higher in the JOL condition than the No-JOL condition (*M*s = 1.22 vs. .69), *t*(144) = 3.82, *d* = .74, *p* < .001, but *d*′ for happy faces did not differ significantly between the JOL and No-JOL conditions (*M*s = 1.18 vs. .98), *t*(144) = 1.55, *d* = .29, *p* = .122 (see Figure 3 Panel A).

Third, the same ANOVA was conducted for response criterion (*C*), which revealed a main effect of study emotion, *F*(1, 144) = 4.32, *MSE* = .44, η_p_^2^ = .03, *p* = .039, and a main effect of stimulus race, *F*(1, 144) = 34.47, *MSE* = .14, η_p_^2^ = .19, *p* < .001 (see Figure 3 Panel B). Participants used a more conservative response criterion for happy than for angry faces (*M*s = .41 vs. .24), and for White faces than for Black faces (*M*s = .45 vs. .20).

Fourth, the same ANOVA for hits reveals that there was a main effect of stimulus race, *F*(1, 144) = 10.19, *MSE* = .02, η_p_^2^ = .07, *p* = .002 and an interaction between participant race and stimulus race, *F*(1, 144) = 8.49, *MSE* = .02, η_p_^2^ = .06, *p* = .004. Overall, hit rates were higher for Black faces than White faces (*M*s = .58 vs. .53). Additionally, Black participants had more hits for Black faces than for White faces (*M*s = .61 vs. .52), *t*(144) = 4.21, *d* = .47, *p* < .001, while White participants had comparable hits for Black and White faces (*M*s = .55 and .55), *t*(144) = .20, *d* = .01, *p* = .840 (see Figure 3 Panel C).

Last, the same ANOVA for false alarms indicates that there were main effects of JOL condition, *F*(1, 144) = 10.22, *MSE* = .04, η_p_^2^ = .06, *p* < .001, of study emotion, *F*(1, 144) = 4.72, *MSE* = .05, η_p_^2^ = .03, *p* = .032, and of stimulus race, *F*(1, 144) = 37.94, *MSE* = .01, η_p_^2^ = .21, *p* < .001. False alarms were lower in the JOL condition than the No-JOL condition (*M*s = .22 vs. .30), for happy than for angry faces (*M*s = .23 vs. .29), and for White than for Black faces (*M*s = .22 vs. .30). There was also an interaction between participant race and stimulus race *F*(1, 144) = 13.96, *MSE* = .01, η_p_^2^ = .09, *p* <.001. White participants had more false alarms for Black faces than for White faces (*M*s = .31 vs. .17), *t*(144) = 7.18, *d* = .83, *p* < .001, while false alarms did not differ significantly across Black faces and White faces (*M*s = .30 vs. .26) in Black participants, *t*(144) = 1.67, *d* = .17, *p* = .097 (see Figure 3 Panel D).

## 5. Discussion

Recently, Guo et al. (2025) observed a novel pattern of emotion-related difference in JOL reactivity for face recognition, such that JOLs produce positive reactivity for negative faces but not for positive faces. While Guo et al. used a mixed list of faces with different emotional expressions, the current study tested whether the emotion-related difference in JOL reactivity also arises in a pure-list design, which offers a critical test of whether the changed-goal hypothesis can account for the finding. Specifically, a mixed-list design is necessary for participants to compare easier and harder item types and thus switch their learning goals based on their metacognitive evaluation of item difficulty. However, a pure-list design offers little opportunity for such comparative processing and thus minimizes the motivation for goal changes. Therefore, the changed-goal hypothesis should predict JOL reactivity in a mixed-list but not in a pure-list design. However, we replicated the finding of positive reactivity for negative faces and null reactivity for positive faces in a pure-list design, which is inconsistent with the changed-goal hypothesis.

Since we reported evidence indicating that the changed-goal hypothesis cannot account for the emotion-related difference in JOL reactivity for face recognition, could other alternative theories account for this novel finding? Another major JOL reactivity theory that is widely studied is the cue-strengthening hypothesis (Soderstrom et al., 2015), which combines the cue-utilization framework (Koriat, 1997) with the transfer-appropriateness principle (Winstanley, 1996). Specifically, the hypothesis suggests that individuals rely on various cues to predict the strength of their memory for the item rather than directly assessing their memory strength (Koriat, 1997), and making JOLs in turn strengthens the cues that inform JOLs. Thus, JOLs should produce positive reactivity if the strengthened cues are diagnostic of subsequent memory performance. This hypothesis is useful for explaining positive reactivity for semantically related pairs in the word-pair paradigm (e.g., Chang & Brainerd, 2023; Halamish & Undorf, 2023; Janes et al., 2018; Maxwell & Huff, 2023; Myers et al., 2020; Soderstrom et al., 2015; Witherby et al., 2023) because the semantic relatedness is a salient cue that informs JOLs, and it is simultaneously a diagnostic memory cue, as semantically related pairs were better recalled than unrelated pairs. 

However, in the current design, although emotional facial expressions clearly inform JOLs, as supported by both Guo et al.’s and our findings, it remains ambiguous whether they are also a diagnostic memory cue. While some evidence shows that positive and negative faces were better recognized than neutral faces (Baudouin et al., 2000; Johansson et al., 2004; Wang, 2013), there have also been several contradictory findings showing that emotional faces were recognized comparably or even worse relative to neutral faces (Hourihan, 2020; Stevens et al., 2025; Van Den Stock & de Gelder, 2014). Therefore, based on the inconclusive findings in the face recognition literature, it remains unclear whether emotionality (positivity or negativity, as opposed to neutral valence) is a diagnostic memory cue. Accordingly, the cue-strengthening hypothesis does not yield a clear prediction regarding emotion-related impacts on JOL reactivity, which limits its explanatory power for the emotional disparity in JOL reactivity. 

Instead, we propose that the elaborative processing account (Chang & Brainerd, 2025) can accommodate these findings. This account proposes that making JOLs enhances the elaborative processing of meaning content more than people spontaneously do. As Chang and Brainerd (2025) demonstrated, even though positive reactivity was repeatedly found for semantically related pairs in associative recall (see Ingendahl et al., 2025 for a meta-analysis), there was consistently no JOL reactivity for phonologically related pairs (e.g., *coarse*–*course*), which lack semantic relations. However, when Chang and Brainerd (2025) used phonological–semantic mediated pairs (e.g., *coarse*–*class*, where the cue word *coarse* is phonologically related to a never-shown mediator *course*, and the mediator *course* is semantically related to the target word *class*) instead of purely phonologically related pairs, they found a positive reactivity just as for semantically related pairs. These findings suggest that the processing enhancement effect of making JOLs is restricted to meaning content rather than relations of any nature. 

Additional support for this account comes from Tekin and Roediger (2020), who examined JOL reactivity in a depth-of-processing paradigm. Those authors found that making JOLs produced positive reactivity for word learning, but the reactivity was attenuated in the deep-processing condition (where participants make judgments about word meaning during encoding) compared to the shallow-processing condition (where participants make judgments about word phonology or orthography during encoding). As Tekin and Roediger discussed, this may be because both JOL-making and deep processing engage participants in semantic elaboration. Thus, making JOLs offered relatively limited additional benefits in the deep processing condition, where elaborative processing is already engaged, but it produces larger memory benefits in the shallow processing condition, where elaborative processing is limited. Therefore, such findings were again in line with the elaborative processing account.

Returning to the finding of different JOL reactivity across positive and negative faces, how could the elaborative processing account explain it? In the face recognition literature, several studies have found that faces were better recognized when encoded with positive rather than negative expressions (D’Argembeau et al., 2003; D’Argembeau & Van der Linden, 2007; Liu et al., 2014), a pattern replicated in the No-JOL condition of the current study. Critically, D’Argembeau and Van der Linden (2007) revealed that the superior recognition for faces encoded with positive expressions lies in recollection rather than familiarity, which is a memory process especially sensitive to elaborative encoding. Thus, D’Argembeau and Van der Linden (2007) concluded that faces with positive expressions engage richer elaborative encoding of the facial identities than those with negative expressions. This interpretation is further supported by other findings showing that negative expressions draw more attention to the expression itself and thus disrupt the processing of other expression-irrelevant but identity-related facial features (Eastwood et al., 2003; Vuilleumier et al., 2001). Taken together, these findings suggest that facial identity tends to be more elaborately processed when encoded with positive expressions relative to negative expressions.

If positive faces are naturally more elaboratively processed than negative faces and making JOLs primarily enhances memory by promoting elaborative processing, then the memory benefits of making JOLs should be more pronounced for negative faces than positive faces. In other words, as positive faces may already engage relatively rich elaboration, making JOLs may provide limited incremental benefits. By contrast, JOLs could be more complementary to the processing of negative faces, for which spontaneous identity-related elaboration is relatively limited. Moreover, based on the elaborative processing account, the difference in reactivity across positive and negative faces should not depend on mixed-list and pure-list designs because the account attributes this difference to how JOLs interact with the spontaneous elaborative processing afforded by the positive and negative faces rather than to participants’ explicit comparison across positive and negative faces. However, we acknowledge that this theoretical explanation is post hoc, and the current study was not designed to directly test this hypothesis. Thus, we would encourage future research to further test this account, such as examining whether manipulating the level of elaboration could moderate JOL reactivity for positive versus negative faces.

It is important to note that the pattern observed in the current study—namely, positive JOL reactivity for positive stimuli but no reactivity for negative stimuli—may be specific to face processing rather than generalizable to other stimulus domains. Based on the elaborative processing account, the emotion-based pattern in reactivity depends on whether positive or negative stimuli are more elaborately processed during encoding, which can vary across stimulus domains. To illustrate, the face-recognition literature has often documented a *positivity bias*, which has been attributed to more elaborative encoding of facial identity when faces display positive rather than negative expressions (D’Argembeau et al., 2003; D’Argembeau & Van der Linden, 2007; Liu et al., 2014). In contrast, the verbal memory literature often reports a *negativity bias* such that negative words were often found to elicit more semantic elaboration than positive words (De Pascalis et al., 2009; Dewhurst & Parry, 2000; Fan et al., 2023; Holt et al., 2009). Accordingly, in terms of verbal memory, the elaborative processing account should make an opposite prediction as for face recognition; that is, if JOLs produce positive reactivity by enhancing elaborative processing, such effects should be weaker for negative words than for positive words. Aligned with this prediction, a recent study found that making JOLs produces positive reactivity in recognition memory for positive and neutral words but not for negative words (Guo et al., 2024). Therefore, the difference in JOL reactivity based on emotional valence appears to depend on stimulus domain, and future research is recommended to examine the emotional disparity in JOL reactivity for other emotional stimuli, such as complex emotional scenes or narratives.

Additionally, it is worth noting that we also replicated the classic CRE that cross-race faces were recognized worse than same-race faces (Meissner & Brigham, 2001; Sporer, 2001; Young et al., 2012). Importantly, consistent with our recent finding (Chang et al., 2026), JOL condition interacted with neither stimulus race nor participant race, suggesting that JOL reactivity did not vary across same-race and cross-race faces. Moreover, by using the same faces with different emotional expressions between study and test, the current finding complements those of Chang et al. (2026) in showing that the positive reactivity for both same-race and cross-race faces was restricted to faces displaying neutral or negative expressions, at least when faces were tested with neutral expressions. Here, the invariance of JOL reactivity across same-race and cross-race faces again runs counter to the changed-goal hypothesis, because same-race faces were perceived as easier to remember than cross-race faces, at least for Black participants, and thus the changed-goal hypothesis should predict positive reactivity for same-race faces but negative reactivity for cross-race faces. However, this finding can again be accounted for by the elaborative processing account, because prior literature has established that elaborative processing improves recognition for both same-race and cross-race faces (Burgess & Weaver, 2003; Devine & Malpass, 1985; Schwartz et al., 2023; Stahl et al., 2010). Accordingly, if positive JOL reactivity is rooted in enhanced elaborative processing, it should improve memory for both same-race and cross-race faces.

Lastly, we would like to acknowledge some limitations of the current study that may potentially motivate future research. Firstly, like most standard JOL reactivity research, the study and test phases in the current study were separated by a brief buffer test, which produced a fairly short retention interval. While the JOL reactivity effect is robust across short and long retention intervals in verbal memory (Witherby & Tauber, 2017), it remains an open question whether the same pattern extends to face recognition. Thus, future studies are recommended to examine whether the present pattern persists after substantially longer retention intervals. Secondly, the data collection for the current study was conducted remotely on Prolific in an unsupervised environment. Although prior research suggests that online testing yields comparable JOL reactivity findings and face recognition performance relative to laboratory testing (Bobak et al., 2023; Germine et al., 2012; Ingendahl et al., 2025; Popovic et al., 2025), we acknowledge that multiple factors, such as distractions, interruptions, and variability in testing conditions, cannot be fully controlled or detected by attention checks in online testing. Future research could therefore replicate the present findings under more controlled laboratory conditions and examine whether they generalize to more naturalistic face-recognition contexts. Thirdly, future research is also recommended to explore individual-difference factors that may affect JOL reactivity for emotional stimuli, such as individual differences in emotion-regulation strategies, baseline emotional state, and metacognitive ability.

## 6. Conclusions

In the current study, we replicated Guo et al.’s (2025) finding of positive JOL reactivity for negative faces but null reactivity for positive faces using a pure-list design. This illustrates that the changed-goal hypothesis cannot fully explain why JOLs produced different reactivity for positive versus negative faces. We proposed that an alternative account, the elaborative processing account, can potentially accommodate the emotion-related difference in JOL reactivity, which posits that making JOLs strengthens elaborative processing of the meaning content more than people spontaneously do. As positive faces tend to be processed more elaboratively than negative faces, there is relatively limited room for JOLs to induce additional elaboration for positive than negative faces. In contrast, negative faces, which are processed relatively less elaboratively, may be more sensitive to the additional elaboration elicited by JOLs and thus more likely to display positive JOL reactivity. Last, we also replicated our recent finding that JOL reactivity did not vary across same-race and cross-race faces, and our findings identify a boundary condition that the positive reactivity for both same-race and cross-race faces is restricted to faces encoded with negative or neutral expressions.

## Data Availability

All relevant data, materials, and codes are available at https://osf.io/uz4ef (accessed on 20 July 2026).
